# Colorectal carcinomas with *KRAS* codon 12 mutation are associated with more advanced tumor stages

**DOI:** 10.1186/s12885-015-1345-3

**Published:** 2015-05-01

**Authors:** Wenbin Li, Tian Qiu, Wenxue Zhi, Susheng Shi, Shuangmei Zou, Yun Ling, Ling Shan, Jianming Ying, Ning Lu

**Affiliations:** Department of Pathology, Cancer Hospital, Chinese Academy of Medical Sciences & Peking Union Medical College, National Cancer Center, Beijing, China

**Keywords:** *KRAS* mutation, *BRAF* mutation, Colorectal cancer, Codon 12 and 13, DNA mismatch repair

## Abstract

**Background:**

*KRAS* mutation occurs in 35%-40% of colorectal cancer (CRC). The aim of our study was to evaluate the pathological and molecular features of specific *KRAS* mutated colorectal carcinomas. *KRAS* and *BRAF*^*V600E*^ mutation tests were performed in 762 primary tumors from a consecutive cohort study of Chinese CRC patients.

**Methods:**

DNA mismatch repair (MMR) status was determined by immunohistochemistry (IHC) staining. Assessment of *KRAS* and *BRAF* V600E mutational status was performed using a multiplex allele-specific PCR-based assay.

**Results:**

Mutations of *KRAS* (34.8%) and *BRAF*^*V600E*^ (3.1%) were nearly mutually exclusive. Both *KRAS*- and *BRAF*- mutated tumors were more likely to be located at proximal colon than wild-type (WT) carcinomas. *KRAS*-mutated carcinomas were more frequently observed in female patients (47.5% *vs* 37.1%, p = 0.005) and mucinous differentiation (34.7% *vs* 24.8%, p = 0.004), but have no difference between lymph node (LN) metastases and among pTNM stages. Whereas, *BRAF*-mutated carcinomas more frequently demonstrated histologic features such as proximal location (60.9% *vs* 20.9%, p = 0.001), low-grade histology (43.5% *vs* 18.0%, p = 0.005), mucinous differentiation (69.6% *vs* 25.9%, p = 0.001) and deficient MMR (dMMR) (21.7% *vs* 7.6%, p = 0.03). In particular, *KRAS* codon 12 mutated carcinomas had increased lymph node metastasis (odds ratio [OR] = 1.31; 95% confidence interval [CI] = 1.04 to 1.65; *P* = 0.02) and were more likely in higher disease stage (III-IV) than that of WT carcinomas (OR = 1.30; 95% CI = 1.03 to 1.64; *P* = 0.03). However, there were no significant differences in lymph node metastasis and disease stage between *KRAS* codon 13 mutated carcinoma and WT carcinoma patients.

**Conclusions:**

In summary, *KRAS* codon 12 mutation, but not codon 13 mutation, is associated with lymph node metastasis and higher tumor stages.

## Background

Colorectal cancer (CRC) is the third most common cancer and the fourth leading cause of cancer mortality in China [1]. CRC is a multistep process based on the accumulation of somatic mutations and can be divided into at least two different and seemingly independent pathways, which is the chromosomal instability (CIN) and microsatellite instability (MSI) pathways [2-5]. CIN occurs in about 85% patients with sporadic CRC and is thought to originate from a relatively uniform and linear accumulation of genetic changes in *APC*, *KRAS* and *TP53* genes [6]. However, sporadic tumors with MSI-high (MSI-H) are originated from promoter hypermethylation of the *MLH1* gene, more frequently found in females, and tend to be poorly differentiated tumors, of mucinous subtype and often harboring somatic mutations in *BRAF*^*V600E*^ [7,8]. Dysfunction of the DNA mismatch repair (MMR) system is the main cause of MSI, which leads to accelerated accumulation of single nucleotide mutations and alterations in the length of simple, repetitive microsatellite sequences [9].

Recently, MMR status, *KRAS* and *BRAF* mutation status have attracted remarkable attention due to their potential prognostic and predictive role in colorectal carcinomas [10-12]. *KRAS* mutations are present in approximately 35% to 40% of colon cancers, with roughly 2/3 of these mutations in codon 12 and 1/3 in codon 13 [11,13]. The presence of a *KRAS* mutation is predictive for resistance to anti-EFGR monoclonal antibodies (mAbs) in advanced colon cancer [13-16]. However, the biological and functional consequences of *KRAS* mutations at codon 12 may be different from those at codon 13 [17-19]. It has been suggested that patients whose tumors harbor a *KRAS Gly13Asp* mutation may benefit from anti-EGFR mAb therapy [20-22]. The clinical significance of *KRAS* mutation in colorectal carcinoma patients is controversial; some studies reported no association with survival, whereas others suggested that patients with *KRAS* mutated colorectal carcinoma have poorer outcome for any mutation subtype, mutation in codon 12 only or codon 13 only [19,21,23,24].

On the other hand, *BRAF* is also involved in the *MAPK/ERK* signaling pathway and oncogenic mutations in this gene have been identified in CRC [25]. Several studies have reported a range of frequencies regarding *BRAF* mutations in colon cancer (7.1%–13.3%), with the most frequent mutation being a single substitution at nucleotide 1,799, substituting valine for glutamic acid (V600E) [7]. Mutations in *BRAF* are most commonly found in microsatellite-unstable (MSI) tumors, whereas they are less common in microsatellite-stable (MSS) tumors [10,26,27]. Mutations in *KRAS* and *BRAF* genes seem to occur in a mutually exclusive manner, and both are suggested as integral components for an effective molecular classification of colorectal cancer.

More accurate prediction of outcome among patients with CRC remains a worthy area of investigation. Although the roles of MMR status, *KRAS* and *BRAF* mutations on clinical outcome are frequently documented, the accurate analysis of these 3 features on clinicopathologic and prognosis with emphasis on the specific *KRAS* gene mutation is still missing. The aim of this study was to evaluate the prognostic role of MMR status, *BRAF* mutations and specific *KRAS* point mutation in 762 patients in Chinese population, and several clinicopathologic features to better stratify colorectal cancer patients.

## Methods

### Study population

The clinicopathological records of 762 patients with corresponding paraffin-embedded material available for molecular analysis were retrospectively collected from the Department of Pathology, Cancer Institute and Hospital, Chinese Academy of Medical Sciences, Beijing, China from December 2011 to December 2012. Patients who had a history of preoperative radiochemotherapy or gastrointestinal surgical resection were excluded. Histopathological criteria were reviewed and included tumor diameter, pT and pN classification, grade of differentiation, histological subtype, tumor location, tumor size as well as the pTNM stage. The size of each tumor was evaluated by measuring its maximum diameter. Grading was determined according to the 2010 WHO histological classification. The pTNM stage system of the 7th edition AJCC cancer staging was used. Evaluating of M stage was mainly according to confirmed pathological results and/or radiological data. Location in the colon was designated as proximal colon for tumors located in the cecum, ascending colon and transverse colon, and as distal colon for tumors in the descending colon and sigmoid colon. Mucinous differentiation in the tumor was defined by the presence of pools of extracellular mucin-containing clusters of carcinomatous cells. When >50% of analyzed tumor demonstrated mucinous differentiation, the tumor was classified as mucinous carcinoma. The study was approved by the Institute Review Board of the Cancer Hospital, CICAMS. Each participant signed an Institutional Review Board approved informed consent in accordance with current guidelines.

### *KRAS* and *BRAF*^*V600E*^ mutation analysis

Assessment of *KRAS* and *BRAF* V600E mutational status was performed in the Molecular Pathology Laboratory of Department of Pathology, CICAMS, using appropriate quality control procedures. Mutation status was determined using genomic DNA extracted from macrodissected formalin-fixed, paraffin-embedded tumor tissue. Both *KRAS* (codons 12 and 13) and *BRAF* (p.V600E) mutation tests were performed using a multiplex allele-specific PCR-based assay (ACCB, Beijing, China), together with the Stratagene Mx3000P (Agilent Technologies Inc, Santa Clara, CA), which assesses seven different potential mutations in *KRAS* codons 12 and 13 (*Gly12Ala, Gly12Asp, Gly12Arg, Gly12Cys, Gly12Ser, Gly12Val,* and *Gly13Asp*). Neither *KRAS* nor *BRAF*^*V600E*^ mutated tumors were designated as WT carcinomas.

### DNA mismatch repair proteins expression

A panel of four-antibody of MMR proteins was performed as a routine practice in our pathological department, contained MLH1, PMS2, MSH2 and MSH6. All of the 762 samples were stained in an autostainer (Autostainer Link 48, Dako, Denmark). Primary mouse monoclonal antibodies included MLH1 antibody (ES05, Dako), MSH2 antibody (FE11, Dako). Primary rabbit monoclonal antibodies included MSH6 antibody (EP49, Dako) and PMS2 antibody (EP51, Dako). Carcinomas were considered as deficient MMR (dMMR) when there was a completely absent staining of a detectable nuclear signal in neoplastic cells for at least one protein. While the adjacent normal mucosa or stromal/lymphoid cells that showed presence of nuclear staining are regarded as internal positive control.

### Statistical analysis

The primary objective of this study was to identify distinct clinicopathologic features associated with specific *KRAS* and *BRAF*^*V600E*^ mutation status. Differences of patient characteristics and clinicopathologic factors in the two-dimensional cross-comparison were evaluated statistically by Pearson’s χ^2^-test or Fischer’s exact test. Statistical tests were two-sided, and *P* < 0.05 were considered significant. Logistic regression models were used to detect associations of these characteristics with each of the specific *KRAS* mutations and provided estimates of odds ratio (ORs) and confidence intervals (CIs). Statistics were carried out using SPSS software (version 16.0 of SPSS, Chicago, IL, USA).

## Results

Primary samples from 762 colorectal carcinoma patients were analyzed for *KRAS*, *BRAF* gene mutations and MMR status. Mutations of *KRAS* occurred in 34.8% of colorectal carcinomas. *BRAF*^*V600E*^ mutation was demonstrated in 3.1% of colorectal carcinomas. There was one tumor demonstrating mutations in both *KRAS* and *BRAF*; this case was excluded from the analysis. Mutated carcinomas were compared with non mutated carcinomas for sex, age, histological features and molecular characteristics (Table 1). In addition, given that non-*KRAS*-mutated tumors include a distinct subset characterized by *BRAF* mutation, analyses were also performed to compare *KRAS*-mutated tumors with both *BRAF*-mutated tumors and the remaining subset of colorectal carcinomas, with observed neither somatic oncogene mutation.

**Table 1:** **Distributions of clinicopathologic characteristics by** ***KRAS*** **and** ***BRAF*** **mutation status**

Characterics	Mutant*KRAS*	Wild-type*KRAS*	*P*-value	Mutant*BRAF*	Wild-type*BRAF*	*P*-value
	(n = 265)	(n = 496)		(n = 23)	(n = 738)	
**Sex**			0.005			0.26
Male	139 (52.5%)	312 (62.9%)		11 (47.8%)	440 (59.6%)	
Female	126 (47.5%)	184 (37.1%)		12 (52.2%)	298 (40.4%)	
**Tumor location**			0.004			<0.0001
Proximal colon	73 (28.3%)	90 (18.7%)		14 (60.9%)	149 (20.9%)	
Distal colon	52 (20.2%)	135 (28.0%)		5 (21.7%)	178 (25.0%)	
Rectum	133 (51.5%)	257 (53.3%)		4 (17.4%)	386 (54.1%)	
**pT stage**			0.39			0.35^‡^
pT1-2	31 (11.7%)	69 (13.9%)		1 (4.3%)	98 (13.3%)	
pT3-4	234 (88.3%)	427 (86.1%)		22 (95.7%)	640 (86.7%)	
**pN stage**			0.09			0.11
pN0	124 (46.8%)	264 (53.2%)		8 (34.8%)	380 (51.5%)	
pN1-2	141 (53.2%)	232 (46.8%)		15 (65.2%)	358 (48.5%)	
**Disease stage**			0.10			0.13
I-II	123 (46.2%)	260 (52.4%)		8 (34.8%)	374 (50.7%)	
III-IV	143 (53.8%)	236 (47.6%)		15 (65.2%)	364 (49.3%)	
**Tumor grade**			0.26			0.005^‡^
G1-2	221 (83.4%)	397 (80.0%)		13 (56.5%)	605 (82.0%)	
G3	44 (16.6%)	99 (20.0%)		10 (43.5%)	133 (18.0%)	
**Histological type**			0.004			<0.0001^‡^
Mucinous	92 (34.7%)	123 (24.8%)		16 (69.6%)	191 (25.9%)	
Ohter	173 (65.3%)	373 (75.2%)		7 (30.4%)	547 (74.1%)	
**MMR status**			0.76			0.03§
Proficient	243 (91.7%)	458 (92.3%)		18 (78.3%)	682 (92.4%)	
Deficient	22 (8.3%)	38 (7.7%)		5 (21.7%)	56 (7.6%)	
**Age, y**			0.65†			0.35^†^
Mean (SD)	57.7 ± 11.3	57.3 ± 11.5		59.6 ± 10.1	57.3 ± 11.4	
Median	58.5	58.0		59.0	58.0	
Range	27.0-84.0	21.0-87.0		38.0-82.0	21.0-87.0	
**Age,y**			0.95			0.23^‡^
<45	37 (14.0%)	70 (14.1%)		1 (4.3%)	105 (14.2%)	
≥45	228 (86.0%)	426 (85.9%)		22 (95.7%)	633 (85.8%)	
**Tumor size (cm)**			0.68†			0.004^†^
Mean (SD)	4.95 ± 2.08	4.89 ± 1.81		5.83 ± 2.13	4.87 ± 1.89	
Median	4.5	4.5		6	4.5	
Range	1.5-17.0	1.0-14.0		3.0-11.0	1.0-17.0	
**Tumor size**			0.32			0.09
<4.5 cm	121 (45.7%)	208 (41.9%)		6 (26.1%)	322 (43.6%)	
≥4.5 cm	144 (54.3%)	288 (58.1%)		17 (73.9%)	416 (56.4%)	

*Abbreviations*: *MMR* mismatch repair, *SD* standard deviation.

^†^Two-sided Kruskal Wallis test.

^‡^Two-sided χ^2^ test with continuity correction.

^§^Fischer’s exact test.

Others are two-sided χ^2^ test.

### Clinical information and morphological characteristics

The mean age at presentation for *KRAS*-mutated carcinoma was 57.7 ± 11.3 years, which was no significantly different to that for non-mutated-*KRAS* carcinoma at 57.3 ± 11.5 years and WT carcinoma cases at 57.1 ± 11.5 years. Furthermore, regarding the age, there was no significant difference in younger (<45) and older (≥45) patients among *KRAS*-mutated, *KRAS*-wild type and *BRAF*-mutated carcinomas.

Gender distribution did not differ significantly between *BRAF*-mutated colorectal carcinomas. However, female patients were more likely to possess *KRAS* mutation than males (47.7% *vs* 37.1%, *P* = 0.004). *BRAF*-mutated carcinomas were more likely to be found in proximal colon than wild-type *BRAF* tumors (60.9% *vs* 20.9%, *P* < 0.0001). In addition, compared with *KRAS*-wild type carcinomas, *KRAS*-mutated carcinomas were more likely located in proximal colon (28.3% *vs* 18.7%, *P* = 0.004). Twenty five subjects had synchronous carcinomas in both proximal colon and rectum, including 6 *KRAS*-mutated and 19 *KRAS*-wild type carcinoma. However, there was no synchronous carcinoma found in *BRAF*-mutated patients in our study. In addition, *BRAF*-mutated carcinomas were significantly associated with larger tumor size compared with wild-type *BRAF* tumors (5.83 ± 2.13 vs 4.87 ± 1.89; *P* = 0.004).

Both *BRAF*- and *KRAS*-mutated carcinomas demonstrated more frequently mucinous differentiation when compared with *BRAF-* and *KRAS-* wild type carcinomas respectively (34.7% *vs* 24.8% and 69.6% *vs* 25.9%; *P* = 0.004 for *KRAS*-mutated carcinomas *vs KRAS*-wild type carcinomas; *P* < 0.0001 for *BRAF*-mutated carcinomas vs *BRAF*-wild type carcinomas). *BRAF*-mutated carcinomas were observed with higher tumor grade (G3) than *BRAF*-wild type carcinomas (43.5% *vs* 18.0%; *P* = 0.005), however, this did not differ significantly with *KRAS*-mutated and -wild type carcinomas. In addition, there were no significantly difference in aspects of pT stage, pN stage and disease stage among *KRAS*-mutated, *BRAF*-mutated and wild type carcinomas.

### Differences with specific *KRAS* mutations in CRC

The distribution and frequencies of the various tumor and clinicopathological characteristics of specific *KRAS* mutation were summarized in Table 2. Mutation frequencies at codon 12 and codon 13 were 26.9% (205/761) and 7.9% (60/761), respectively. The most common variant in codon 12 was the *p.G12D/A* mutation (101/265, 38.1%), followed by the *p.G12V* mutation (64/265, 24.2%). The *p.G13/D* mutation frequency was 22.6% (60/265). There was no gender preponderance between codon 13 mutation and WT carcinoma patients. However, female patients were more likely to carry codon 12 mutations than WT carcinoma cases (47.8% *vs* 36.4%; OR = 1.38; 95% CI = 1.10 to 1.74; *P* = 0.005). Codon 12 and 13 mutated carcinomas were more likely to be found in the proximal location than WT carcinomas respectively (Codon 12: 29.1% *vs* 16.7%; OR = 3.17; 95% CI = 2.24 to 4.49; *P* < 0.0001. Codon 13: 25.0% *vs* 16.7%; OR = 3.37; 95% CI = 1.67 to 6.82; *P* < 0.0001). Both codon 12 and codon 13 mutated carcinomas demonstrated more frequently mucinous differentiation when compared with WT carcinomas (Codon 12: 33.7% *vs* 21.1%; OR = 1.53; 95% CI = 1.21 to 1.93; *P* = 0.001; Codon 13: 36.7% *vs* 21.1%; OR = 1.95; 95% CI = 1.20 to 3.17; *P* = 0.001).

**Table 2:** **Distributions of clinicopathologic characteristics by** ***KRAS*** **codon 12 and codon 13 mutation**

Characterics	Mutant*KRAS*Codon 12	*Null carcinoma*	*P*-value (Codon 12*vs Null*)	Mutant*KRAS*Codon 13	*P*value (Codon 13*vs Null*)
	(n = 205)	(n = 473)		(n = 60)	
**Sex**			0.005		0.12
Male	107 (52.2%)	301 (63.6%)		32 (53.3%)	
Female	98 (47.8%)	172 (36.4%)		28 (46.7%)	
**Tumor location**			<0.0001		<0.0001
Proximal colon	58 (29.1%)	76 (16.7%)		15 (25.0%)	
Distal colon	40 (20.1%)	253 (55.7%)		13 (21.7%)	
Rectum	101 (50.8%)	125 (27.6%)		32 (53.3%)	
**pT stage**			0.20		0.89
pT1-2	22 (10.7%)	68 (14.4%)		9 (15.0%)	
pT3-4	183 (89.3%)	405 (85.6%)		51 (85.0%)	
**pN stage**			0.02		0.90
pN0	91 (44.4%)	256 (54.1%)		33 (55.0%)	
pN1-2	114 (55.6%)	217 (45.9%)		27 (45.0%)	
**Disease stage**			0.03		0.99
I-II	90 (43.9%)	252 (53.3%)		32 (53.3%)	
III-IV	115 (56.1%)	221 (46.7%)		28 (46.7%)	
**Tumor grade**			0.14		0.26^‡^
G1-2	176 (85.9%)	384 (81.2%)		45 (75.0%)	
G3	29 (14.1%)	89 (18.8%)		15 (25.0%)	
**Histological type**			0.001		0.007
Mucinous	69 (33.7%)	100 (21.1%)		22 (36.7%)	
Ohter	136 (66.3%)	373 (78.9%)		38 (63.3%)	
**MMR status**			0.87		0.30^‡^
Proficient	190 (92.7%)	440 (93.0%)		53 (88.3%)	
Deficient	15 (7.3%)	33 (7.0%)		7 (11.7%)	
**Age, y**			0.59^†^		0.47^†^
Mean (SD)	57.6 ± 11.4	57.1 ± 11.5		58.3 ± 10.6	
Median	59.0	58.0		57.5	
Range	27.0-84.0	21.0-87.0		31.0-77.0	
**Age,y**			0.86		0.09
<45	31 (15.1%)	69 (14.6%)		4 (6.7%)	
≥45	174 (84.9%)	404 (85.4%)		56 (93.3%)	
**Tumor size (cm)**			0.27		0.96
Mean (SD)	4.99 ± 2.13	4.82 ± 1.76		4.83 ± 1.92	
Median	4.5	4.5		4.4	
Range	1.5-17.0	1.0-14.0		3.5-12.0	
**Tumor size**			0.77		0.28
<4.5 cm	90 (43.9%)	202 (42.7%)		30 (50.0%)	
≥4.5 cm	115 (56.1%)	271 (57.3%)		30 (50.0%)	

*Abbreviations*: *MMR* mismatch repair, *SD* standard deviation, *Null* neither *KRAS* nor *BRAF*^*V600E*^ mutation.

^†^Two-sided Kruskal Wallis test.

^‡^Two-sided χ^2^ test with continuity correction.

Others are two-sided χ^2^ test.

**Figure 1:**
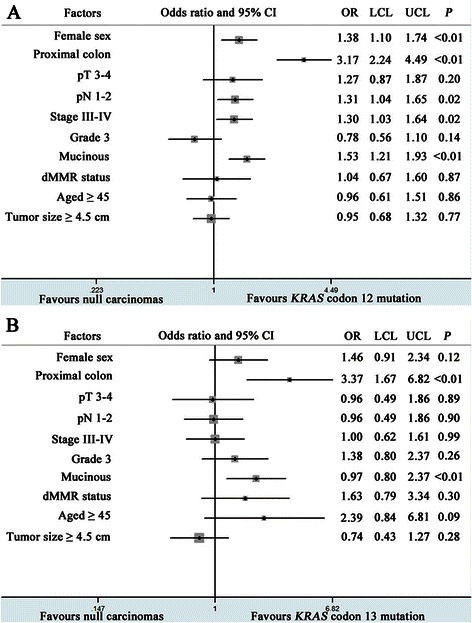
Forest plot of univariate logistic model associations with *KRAS* codon 12 mutation status **(A)** and codon 13 mutation status **(B)**. P values are for two-sided Pearson χ^2^ test. CI = confidence interval; dMMR = deficient mismatch repair; LCL = lower confidence limit; UCL = upper confidence limit; OR = odds ratio.

Univariate logistic regression models identified the following factors as statistically significantly between *KRAS* codon 12 mutated carcinomas and WT carcinomas: gender, pN stage, pTNM stage and histological subtype (Figure 1A). In particular, codon 12 mutated carcinomas had increased lymph node metastasis (pN stage) than WT carcinomas (55.6% *vs* 45.9%; OR = 1.31; 95% CI = 1.04 to 1.65; *P* = 0.02). Moreover, codon 12 mutated carcinomas were more likely in higher disease stages (III-IV) than that of WT carcinomas (56.1% *vs* 46.7%; OR = 1.30; 95% CI = 1.03 to 1.64; *P* = 0.02; *P* = 0.03). However, there were no significant differences in lymph node metastasis and disease stage between codon 13 mutated carcinomas and WT carcinoma patients (Figure 1B). In addition, *KRAS* mutated carcinomas demonstrated no differences in tumor invasion depth (pT stage) with WT carcinomas in both codon 12 and 13 mutated cases.

## Discussion

To the best of our knowledge, this is the first study to demonstrate that patients with *KRAS* codon 12 mutated colorectal carcinomas had a more advanced tumor stage than those with tumors harboring *p.G13D* mutation or wild-type *KRAS* in a large cohort of Chinese population. In addition, the *KRAS* codon 12 mutated carcinomas in our study also displayed morphologic features typically associated with adverse behavior regardless of tumor location. Our data are consistent with previous reports, indicating that the presence of a mutation in *KRAS* codon 12 confers substantially greater oncogenic potential than codon 13 mutation [18,20,28-30].

The incidence of *KRAS* mutation in our reports was approximately 34.8%, which is similar to that described in other studies. However, the frequency of *BRAF*^*V600E*^ mutation was 3.1%, which is lower than in studies of western countries, but similar to that of Asian populations [27,31-33]. This indicates that ethnicity and/or environmental factors might contribute to the discrepancy. Unlike reports from Caucasians, patients with *BRAF*^*V600E*^ mutated tumors in our study did not differ significantly in aspects of sex and age [10]. *KRAS* mutated cases were more likely to be female patients but with no significant difference in age distribution. Several studies reported that *BRAF*^*V600E*^ mutated tumors were more likely to have a more aggressive biology with four or more positive lymph nodes and higher pTNM stage in large population-based cohort studies [34-37]. However, this observation was inconsistent with our results, which presented that mutation in either *BRAF*^*V600E*^ or *KRAS* was not related to the adverse clinicopathological features accompanied by advanced lymph node metastasis and III-IV disease stages. Admittedly, this difference may be related to our smaller size of the *BRAF*^*V600E*^ mutation subgroup. However, both *BRAF*^*V600E*^ and *KRAS* mutated carcinomas were more likely to be located in the proximal colon and mucinous carcinomas. In addition, MMR deficient status was less likely to be associated with *KRAS* mutations, but have a positive correlation with *BRAF*^*V600E*^ mutations [10,35]. Goldstein J, et al. reported that *BRAF*^*V600E*^ mutation is a poor prognostic factor in metastatic MSI-H colorectal tumors [38].

To our knowledge based on the literature search in Pubmed, this is the first study to address the clinicopathological difference between *KRAS* codon 12 and 13 mutations in over 500 of *BRAF*-wild type colorectal cancers in Chinese population [39-41]. Although several previous studies had distinguished the difference between the prognostic associations of *KRAS* mutations in codon 12 and 13, none of the large studies (with a sample size more than 300) controlled for *BRAF* mutation status in their analyses, and results were conflicting [12,24,42,43]. The initial studies considered both mutations including codon 12 and 13 as a whole to analyze the clinicopathological features and disease outcome. Only small and very recent detailed reports assessed the effect of *KRAS* mutations where the codon 12 and 13 were analyzed separately [18,19,21]. RASCAL study, the initial data from clinical trials, suggested that *KRAS* mutation status is an important prognostic factor for progression and outcome in the CRC patients and glycine to valine in codon 12 convey a more aggressive biological manner [22,43,44]. The results have been broadly accepted all over the world except that the patients enrolled in the study were distributed over 21 different countries and the results may be confounded with the different ethnic and environmental factors.

In our study, compared with WT carcinoma cases, both *KRAS* codon 12 and codon 13 mutations were associated with proximal (*vs* distal) tumor site of the colon. The distribution of *KRAS* codon 12 and 13 mutations did not differ considerably by tumor subsite , consistent with findings from other reports [19]. Besides, proximal colon carcinomas were more likely than distal carcinomas to be *KRAS*-mutated and *BRAF*-mutated. Rosty. C, *et al.* reported that both *KRAS*- and *BRAF*-mutated carcinomas more frequently demonstrated focal or predominant mucinous differentiation than WT carcinomas, which was consistent with our findings [11]. In addition, mucinous tumors were more likely than other type of colorectal carcinomas in either *KRAS* codon 12 or 13 mutated tumors. Mucinous colonic adenocarcinoma is a frequently encountered histologic subtype of colorectal tumors, which is often associated with worse clinical outcome and decreased overall survival [45,46].

The most valuable finding of this study is that *KRAS* codon 12 mutated tumors demonstrated more positive lymph nodes and pTNM III-IV stage of disease than WT carcinoma patients, whereas tumors with codon 13 mutation did not differ by number of positive lymph nodes or pTNM stage. This was consistent with findings from a smaller report, which *KRAS* codon 12 mutation was found to be linked with more aggressive clinicopathological features and worse clinical outcomes [19,47]. Mutations in codons 12 and 13 lead to alterations in encoded amino acids adjacent to the GTP binding pocket and reduced the GTPase activity of *KRAS* protein after guanine nucleotide activating protein (GAP) binding [17,48-50]. Subsequently, these conformational and structural changes of the EGFR signaling pathway are out of control with constitutive activation of KRAS protein. Nonetheless, theses structural modifications will be different in case of each codon and the amino acid changed conferring variable activated KRAS effects. In particular, in *vitro* and in *vivo* studies indicate that *KRAS* codon 12 mutations have greater transforming capacity when compared with codon 13 mutations [23,51,52]. On the basis of protein computational analysis, codon 12-mutated *KRAS* remains in an active GTP-bound state longer than codon 13-mutated or wild type *KRAS*. It seems that mutations in codon 13 share the similar protein confirmation with wild type *KRAS* [53]. Consequently, codon 13 mutations confer reduced transforming ability in colon tumor cells.

Despite these positive findings, our study had some limitations. First, because our study lacked data on tumor CIMP (Cpg Island Methylator Phenotype) status and cause of dMMR status, we could not distinguish the correlations of somatic and germline mismatch repair mutations with *KRAS* and *BRAF* mutations. Second, although based on a large population size, the low incidences of *BRAF* mutation (3.1%) made the subgroups relatively small and further validations with more population are needed. Finally, we did not examine other less common mutations in *KRAS* codons 61, 117 and 146, which are also the negative predictive marker for response to anti-EGFR therapy [54].

## Conclusions

In conclusion, our study suggests that specific epidemiologic and clinicopathologic characteristics were associated with *KRAS* and *BRAF*^*V600E*^ mutations in a large cohort of Chinese colorectal carcinoma population. Specifically, tumor size of 6 cm or longer, low-grade histology, and dMMR status were associated with a higher incidence of *BRAF*^*V600E*^-mutated tumors. Both mutations tend to be proximal tumor location and mucinous histology, but *KRAS*-mutated tumors are more common in female patients. Finally, *KRAS* codon 12 mutation, but not codon 13 mutation, is associated with more positive lymph nodes and higher pTNM stages. Because of its correlation with a more advanced stage, patients with *KRAS* codon 12 mutations may have worse survival than those with *KARS* 13 mutations or wild-type *KRAS*. Further studies need to define the mechanism by the clinicopathologic and epidemiologic characteristics that may explain the association with these specific mutations.

## Abbreviations

CRC: Colorectal cancer
MMR: Mismatch repair
IHC: Immunohistochemistry
OR: Odds ratio
CIN: Chromosomal instability
MSI: Microsatellite instability
CIMP: Cpg island methylator phenotype
